# Repurposing Non-Infectious Therapeutic Agents to Aid in the Treatment of Chronic Biofilm Infections

**DOI:** 10.3390/medsci14020226

**Published:** 2026-04-30

**Authors:** Lila Berle, Yash Sodhi, Poonam Mathur, Nazary Nebeluk, James B. Doub

**Affiliations:** 1 University of Maryland School of Medicine, Baltimore, MD 21201, USA; lila.berle@som.umaryland.edu; 2 The Doub Laboratory of Translational Bacterial Research, University of Maryland School of Medicine, Baltimore, MD 21201, USA; nnebeluk@som.umaryland.edu; 3 University of Maryland, College Park, MD 20742, USA; 4 Division of Clinical Care and Research, Institute of Human Virology, University of Maryland School of Medicine, Baltimore, MD 21201, USA; pmathur@ihv.umaryland.edu

**Keywords:** biofilm, chronic infections, ethyl pyruvate, N-acetylcysteine, EDTA, methylene blue

## Abstract

Antibiotics primarily exert their effect on planktonic microbial states, limiting their ability to eradicate biofilms commonly seen in chronic infections. This is because the minimal inhibitory concentration of antibiotics needed to kill microbes in biofilms can be up to 1000 times greater than when microbes are in their planktonic state. Yet up to 70% of all chronic infections are associated with a biofilm component. Consequently, novel therapeutics are needed to aid in the treatment of chronic biofilm infections. One such approach is to repurpose drugs that have demonstrated safety for non-infectious clinical indications. The main advantage of this approach is that the agents have already been shown to be safe for human administration, which can expedite clinical trial development of agents for biofilm infections. Unfortunately, most clinicians are unaware of the antimicrobial properties of some commonly used drugs. Thus, the aim of this Perspective was to discuss the potential of four drugs that have theoretical promise as adjuvants in the treatment of chronic biofilm infections. This was accomplished by providing detailed discussion of each agent with respect to current clinical use, potential mechanisms of antimicrobial activity, and present data to support use as adjuvant biofilm agents.

## 1. Introduction

Antibiotics have revolutionized the treatment of infectious diseases in the 20th century, dramatically increasing the average human lifespan [1]. However, antibiotics primarily exert their effect on planktonic microbial states, limiting their ability to eradicate biofilms commonly seen in chronic infections. These shortcomings are not due to a lack of chemical antimicrobial activity but rather due to pharmacokinetic and pharmacodynamic (PK/PD) factors that prevent effective exposure of microbes to drugs within the biofilm. Biofilms impose unique barriers including restricted diffusion through the extracellular polymeric substance, sequestration of antimicrobials within their matrix, steep metabolic gradients, and a predominantly non-replicating microbial state [2], all of which can cause the minimal inhibitory concentration (MIC) of antibiotics needed to kill microbes in biofilms to be up to 1000 times greater than when microbes are in their planktonic state [3]. Consequently, it is not clinically feasible to dose antibiotics at concentrations necessary to eradicate biofilm microbes due to the potential for significant toxicities. Furthermore, while some antibiotics such as rifamycin have known antibiofilm activity, especially when used in combination with other antibiotics, the ability to fully eradicate in vivo biofilms is not well defined [4].

Unfortunately, it is estimated that up to 70% of all chronic infections have a biofilm component [5]. Curing a biofilm infection requires removal of the implanted material containing adherent biofilms. For some infections, this is easily done by a simple removal of an external device, like a catheter, but for deep-seated infections like prosthetic joint infections, removal of the infected material increases morbidity and mortality and ensues enormous health care costs [6]. The lack of less invasive and risky strategies for the treatment of biofilms in chronic infections highlights a crucial challenge in modern medicine and exposes a major shortcoming of antimicrobial therapies. Therefore, it is paramount to consider new strategies for the treatment of biofilms in chronic infections.

One such strategy is to repurpose drugs that have demonstrated safety and received FDA approval for non-infectious clinical indications. This approach has been proposed to aid in the treatment of antimicrobial resistance but also could be used for the treatment of chronic biofilm-associated infections [7,8]. The main advantage of this approach is that the agents have already been shown to be safe for human administration, which can expedite clinical trial development of agents for biofilm infections [9]. Unfortunately, most clinicians are unaware of the antimicrobial properties of these agents, due to the different clinical context they encounter them in.

Therefore, the aim of this Perspective is to address this knowledge gap and discuss four agents (ethyl pyruvate, N-acetylcysteine, EDTA and methylene blue) that have theoretical promise as adjuvants in the treatment of chronic biofilm infections. While there are other agents (metformin, statins, and NSAIDs to name a few) that also have antibiofilm activity [10,11,12,13], these four agents were chosen based on their broad antimicrobial activity and potential use clinically for commonly encountered biofilm infections [14]. Detailed discussion of each agent with respect to current clinical use, potential mechanisms of antimicrobial activity, and potential PK/PD feasibility in clinically relevant contexts is conducted. Also, data to support use as adjuvant biofilm agents is presented in addition to discussions on how these agents can be used clinically.

## 2. Repurposed Agents

### 2.1. Potential Topical Agents

#### 2.1.1. Methylene Blue

Methylene blue (MB), also known as methylthioninium chloride, is an oxidation–reduction agent approved by the FDA for the treatment of methemoglobinemia in both adult and pediatric patients [15]. It is also an intraoperative and diagnostic additive used to assess lymphatic drainage, surgical leak testing and staining of biofilms [16,17,18]. Common off-label uses include ifosfamide-induced encephalopathy and cyanide toxicity [16]. Overall, studies have shown that MB is safe in systemic concentrations of up to 4 mg/kg. It should be noted that MB acts as a potent reversible inhibitor of monoamine oxidase A (MAO-A), and at higher concentrations, MAO-B. Therefore, when used concurrently with serotonergic medications there is a theoretical risk of inducing serotonin syndrome [19].

Beyond its use in the conditions mentioned above, there are several mechanistic actions that make MB a potential anti-infective. First, MB can inhibit soluble guanylate cyclase and modulate nitric oxide (NO)/cGMP signaling to treat vasoplegia in sepsis models and alter host–pathogen interactions that are mediated by nitric oxide [20]. Second, MB is a photosensitizer, producing ROS and singlet oxygen that can cause irreversible damage to microbial membranes, nucleic acids, and proteins when activated by specific wavelengths of light [21,22]. In vitro and ex vivo studies demonstrate that MB, when exposed to red light (660 nm wavelength), can kill fungi, Gram-negative and positive bacteria, and enveloped viruses [23]. Furthermore, MB has also been shown to have synergistic activity with antibiotics [23,24]. The antimicrobial activity is also light-independent, through multiple mechanisms such as ROS-mediated killing, host modulation, and direct virucidal action [21,22,23,24]. This activity is not limited to bacterial and fungal pathogens and is also pertinent to viral pathogens like H1N1 and SARS-CoV-2 [23].

**Figure 1:**
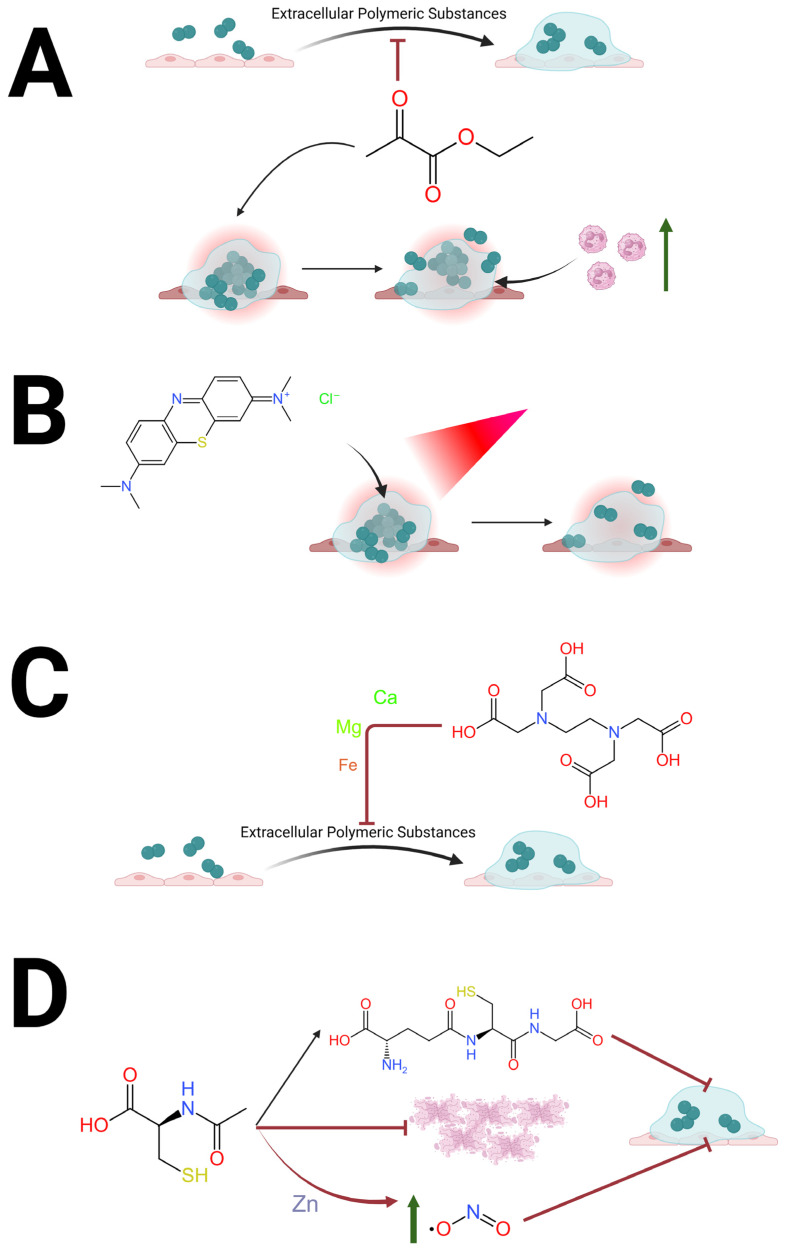
Schematic diagram showing antibiofilm mechanisms for the four repurposed drugs discussed. (**A**) Ethyl pyruvate has the ability to inhibit microbial biofilm formation and decrease concentrations of bacteria in biofilms via disruption of microbial metabolic pathways [46]; (**B**) methylene blue with the addition of red light (660 nm) can decrease the concentrations of bacteria in biofilms via production of reactive oxygen species that cause oxidative damage to microbial cells [23]; (**C**) EDTA can chelate cations (such as calcium, magnesium and iron) needed for stability of the extracellular polymeric substance thereby destabilizing bacterial biofilms [32]; (**D**) N-acetylcysteine acts to destabilize biofilms by chelating zinc leading to accumulation of nitrogen dioxide [55], degrading extracellular DNA in neutrophil extracellular traps [57] and metabolizing to glutathione, thereby decreasing biofilm viscosity [56]. Created in BioRender. Nebeluk, N. (4 April 2026). https://BioRender.com.

MB also has the ability to stain biofilms as well as reduce bacterial CFUs within them [25]. The antimicrobial activity of MB is therefore most pronounced when applied to biofilms and exposed to an external light source [26,27] visualized in Figure 1B. Moreover, MB’s greatest translational potential lies in localized application and antibiofilm concentrations demonstrated in vitro are readily achievable through topical application, avoiding systemic PK constraints and minimizing toxicity concerns [15,16,18]. Photodynamic activation allows for a unique PD profile as instead of relying on intracellular accumulation of a molecule, light-activated MB produces local ROS which have antimicrobial activity against the metabolically quiescent biofilm [21,22]. While the requirement for external light adds complexity to MB’s potential as an adjuvant therapeutic, it could be incorporated into treatment of surgical wound infections, diabetic foot wounds, and intraoperatively in procedures that have a “down time” to allow for MB to dwell on the biofilm while exposed to red light. One such application could be orthopedic infections when infected prosthetics and hardware are removed and new hardware is placed, since there is a significant period between removal of the infected prosthesis and placement of new hardware. The utility and feasibility of using MB for these infections would need to be demonstrated first in translational studies to prove efficacy and then in proof-of-concept trials to show feasibility, but MB’s translational potential with limited side effects further supports its development as an adjuvant antibiofilm agent.

#### 2.1.2. Ethylenediaminetetraacetic Acid (EDTA)

Ethylenediaminetetraacetic acid (EDTA) has multiple diverse clinical applications. It has been used as a chelator for heavy metal toxicity and in lock therapy for prophylaxis against catheter infections [28,29,30]. It has even been evaluated as a possible cardioprotective agent after myocardial ischemia [31]. Even though its cardioprotective effects have not been verified in subsequent trials it was shown that EDTA can be safely systemically administered at doses as high as three grams [31].

The antimicrobial mechanism of action for EDTA has not been fully elucidated, but the leading theory is that EDTA exerts bactericidal and bacteriostatic effects on Gram-negative bacteria by chelating cations necessary for stabilization of the lipopolysaccharides in their outer membrane [32]. This effect has been shown across several species including *Pseudomonas aeruginosa*, *Escherichia coli* and *Proteus mirabilis* [32]. For Gram-positive bacteria that lack robust outer membranes, it is thought that EDTA chelates cations necessary for cell proliferation or acts on an intracellular target [32]. This poorer activity against Gram-positive bacteria has been attributed to these bacteria expressing teichoic acid that competes with EDTA for cation chelation [32].

EDTA’s antimicrobial effects are not limited to planktonic bacteria. Rather, EDTA has exhibited the ability to destabilize preformed biofilms of both Gram-positive and Gram-negative bacteria [33,34,35]. The mechanism behind biofilm destabilization is thought to be secondary to EDTA chelating cations which are important for stabilization of extracellular polymeric substances [33,34,35], visualized in Figure 1C. EDTA is also thought to chelate free calcium and iron, which are essential for cellular adhesion and biofilm viscosity [34,35]. In a study evaluating 20 hemodialysis catheters, EDTA decreased viable cell counts in previously formed biofilm by 50% after 6 h, with no viable cells present in most catheters after 24 h of EDTA exposure, at 40 mg/mL for both Gram-negative and Gram-positive bacteria [34]. In another study, 50 mM (18.6 mg/mL) EDTA significantly decreased *P. aeruginosa* and *S. aureus* biofilms [33]. In one study, the minimal dose to cause toxic effects in animals was 750 mg/kg per day [35], which in an average United States adult weighing 89 kg [36] and having 5.5 L of blood would correspond to a blood concentration of roughly 12.1 mg/mL. Therefore, while the effective antibiofilm concentrations reported in vitro exceed that which is achievable via systemic administration, this reinforces its utility for localized exposure. In clinical cases such as dwell-based catheter lock therapy or impregnation of devices surfaces, the agent can achieve high local concentrations, causing prolonged chelation-mediated destabilization of the extracellular polymeric matrix, with minimal systemic absorption [33,34,35]. This apparent disparity between in vitro effective concentrations and the PK limits of systemic dosing does not preclude translational application, but rather highlights the importance of optimizing delivery.

### 2.2. Potential Systemic Agents

#### 2.2.1. Ethyl Pyruvate

Ethyl pyruvate (EP) is not only a common food additive but also an antioxidant that can protect against ischemic cell damage [37,38]. These effects were first demonstrated in murine models evaluating EP’s benefit in conditions such as mesenteric ischemia, myocardial infarction, hypoxic brain injury and pancreatitis [38,39,40]. EP is thought to exert its effects on the GAS6/Axl pathway, by decreasing lactate dehydrogenase, reactive oxygen species (ROS) and ultimately NLRP-3 inflammasome production. Attenuation of the NLRP-3 inflammasome protects tissues against hypoxic reperfusion injury [41,42,43]. Successes in murine models inspired a phase II clinical trial in humans evaluating the role of EP in preventing hypoxic organ injury in patients on cardiopulmonary bypass. Although there was no significant clinical benefit observed, the studies showed that no toxicity from EP was seen at doses of up to 90 mg/kg [39].

Beyond its antioxidant effects, EP also has anti-inflammatory properties that can reduce aberrant responses to endotoxemia [44]. This has been shown to occur through decreases in production of inflammatory cytokines (IL-1α and IL-1β), thereby limiting host tissue damage [45]. Surprisingly, EP also has direct antimicrobial activities. While multiple antimicrobial mechanisms have been proposed, one prominent hypothesis is that EP interferes with glycolysis and para-glycolytic pathways, inducing microbial toxicity through intracellular ATP depletion and methylglyoxal accumulation [44,46]. EP’s microbial toxicity allows it to have a broad spectrum of antimicrobial activity against yeast and Gram-positive and Gram-negative bacteria with minimal inhibitory concentrations as low as 25 mM or 2.9 mg/mL [44,46].

EP has been proven to directly inhibit biofilm formation and reduce pathogen concentrations within biofilms, visualized in Figure 1A. An example of this can be seen in a study which showed EP almost completely inhibited *Candida* biofilm formation in a dose-dependent manner [46]. In contrast, amphotericin B at concentrations over double its MIC (0.38 μg/mL) was unable to inhibit biofilm formation [46]. Furthermore, 50 mM concentrations of EP decreased the number of Gram-positive and Gram-negative CFUs within preformed biofilm by 3 logs, while conventional antibiotics only minimally decreased the number of CFUs [46]. This suggests EP has antimicrobial activity against not only planktonic bacteria but also the sessile bacterial states seen in biofilms. Unfortunately, there is a paucity of data evaluating EP used in combination with antibiotics to assess synergy for planktonic or biofilm states and this will be needed to advance this therapeutic as an adjuvant antimicrobial agent. While more research is undoubtedly needed, the safety seen in clinical trials using large doses of EP (90 mg/kg) suggests that some therapeutically meaningful tissue-level exposure would be achievable in inflamed or hypoxic microenvironments, such as a chronic biofilm infection [39]. This is important because the antimicrobial concentrations of ethyl pyruvate reported in vitro are higher than the plasma levels achieved in vivo [39]. This discrepancy underscores the importance of distinguishing between direct antimicrobial MIC thresholds and in vivo PD effects. EP’s ability to alter microbial energy metabolism and host signaling pathways provides adjuvant antimicrobial effect even without plasma concentrations corresponding to MIC values [44,46].

#### 2.2.2. N-Acetylcysteine

N-acetylcysteine (NAC) is a widely used antioxidant agent in clinical medicine since it reduces disulfide bonds, acts as a scavenger of ROS, and is a precursor of glutathione synthesis. NAC is the gold-standard treatment of acetaminophen-induced liver toxicity, especially when administered within 10 h of ingestion [45,47]. It also is widely used as a mucolytic agent, since studies have shown it significantly decreases sputum viscosity and expectoration [48,49,50]. Moreover, NAC has been proposed to be a protective agent against contrast-induced nephropathy. Although recent studies have shown no observable clinical benefits, doses as high as three grams per day have been reported to be safe [51,52,53].

NAC also has substantial antimicrobial activity by disrupting thiodisulfide oxidoreductases, a group of proteins that regulate the redox state in Gram-negative bacteria. NAC is also a zinc chelator with significant activity against *P. aeruginosa*. Additionally, when administered with meropenem, NAC synergistically reduces the fractional inhibitory concentration index to 0.5 or below to treat carbapenem-resistant *Klebsiella pneumoniae* [54]. This synergistic activity is also seen when NAC is administered with ampicillin/sulbactam to treat carbapenem-resistant *Acinetobacter baumannii* [54]. NAC also inhibits the growth of Gram-positive bacteria: in an in vitro study of MRSA strains exposed to 30 mM (4.9 mg/mL) NAC, bacterial growth was reduced by 95%. However, once NAC was removed, bacterial growth resumed.

NAC antimicrobial activity is not limited to planktonic microbes; NAC also exhibits activity against preformed biofilms and prevents biofilm formation, via several proposed mechanisms which are visualized in Figure 1D. One is the ability for NAC to be rapidly metabolized to glutathione, and the lytic activity of the thiol group works to thin mucus and decrease biofilm viscosity [55,56,57]. Second, NAC has the ability to degrade extracellular DNA, destabilizing biofilms [55,56,57]. NAC is intrinsically acidic and causes protonation of the phosphodiester bonds in DNA leading to random single- and double-strand breaks [50]. These are further exacerbated by its redox-active thiol group which destabilizes the disulfide bonds with proteins that stabilize the matrix [58]. Lastly, since NAC is a zinc chelator it can inhibit denitrification in anaerobic environments (such as deep regions of biofilms) allowing increased concentrations of nitrogen dioxide to accumulate and prevent further biofilm formation [55]. By decreasing the enmeshment of the biofilm, NAC administration exposes the bacteria within biofilms rendering them more accessible to immunological surveillance [57]. While the concentration of NAC reported in vitro exceeds the steady-state plasma concentrations achieved with oral dosing, the molecule’s PD activity within biofilms is not limited to direct bactericidal effects [54,55,56]. These antibiofilm effects can occur even at concentrations below classical MIC thresholds which rely on direct exposure of microbes to the drug and do not account for the complex in vivo environment of infections [55,56,57].

## 3. Future Directions

We are in the midst of an antimicrobial resistance crisis due to the prolonged overuse of antibiotics. Chronic biofilm infections notoriously spur prolonged courses of antibiotics given the limited ability of conventional antimicrobial agents to degrade biofilms. Therefore, not only does the lack of effective treatment options worsen individual morbidity from biofilm infections but it also exacerbates the antimicrobial resistance crisis. The agents discussed in this Perspective may offer some promise to better control and/or aid in biofilm eradication. These agents already have preexisting safety data, making them attractive agents for clinical development to reduce the morbidity and mortality of chronic biofilm infections. Future translational studies must address the PK/PD feasibility of these agents within the biofilm environment—both delivery and efficacy—in this unique biological niche. Crucial research that will help formulate effective therapeutic approaches includes defining tissue-level drug concentrations at sites of infection, assessing penetration or degradation of extracellular polymeric substance, and determining safety of efficacious exposure concentrations. Integrating PK-informed delivery strategies with mechanistic biofilm studies will be critical to validating the clinical applicability of repurposing these agents as adjuvant therapies.

**Figure 2:**
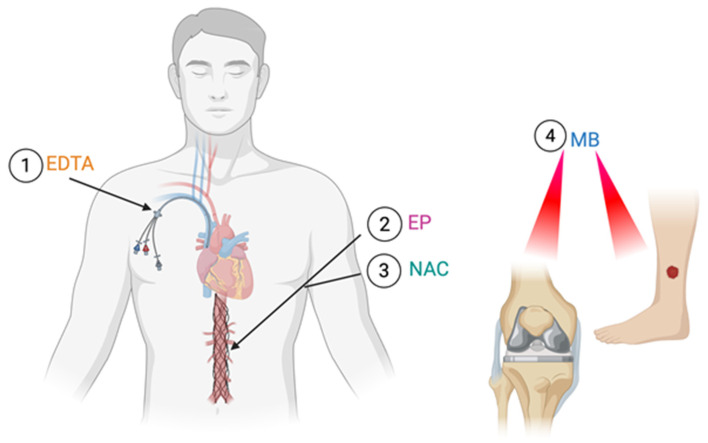
Diagram showing potential biofilm infection indications for the four repurposed drugs. (1) EDTA shows promise as an agent that can be instilled in areas where biofilms are present like catheters for lock therapy. (2,3) Agents such as N-acetylcysteine and ethyl pyruvate have shown safety with high systemic doses and thus can potentially be used to aid in the treatment of systemic or deep-seated biofilm infections such as vascular graft shown here. (4) Methylene blue shows promise as an agent used in combination with red light (660 nm) for wounds and orthopedic infections. Created in BioRender. EDTA: ethylenediaminetetraacetic acid; EP: ethyl pyruvate; NAC: N-acetyl cysteine; MB: methylene blue. Created in BioRender. Mathur, P. (31 March 2026). https://BioRender.com.

Each agent has unique attributes that allow it to be used and administered in different clinical scenarios (Figure 2), which are summarized in Table 1. MB has a broad spectrum of activity to treat polymicrobial infections and has the most potential for efficacy when co-administered with red light, so it could be used to treat wound and prosthetic joint infections. On the other hand, EDTA has been shown to be most effective with prolonged dwell times, so it could be used for indwelling catheter lock therapy and on impregnated or implanted devices to reduce biofilm formation. Lastly, agents like EP and NAC could be administered systematically in combination with antibiotics to treat endovascular or deep-seated chronic biofilm infections.

It is without a doubt that more preclinical and then proof of concept studies are needed for these agents. This is especially the case for studies evaluating synergistic activity with antibiotics, biofilm-associated tolerance and persistence and impact on antimicrobial resistance mechanisms (e.g., efflux activity, quorum sensing). Therefore, while this Perspective’s aim is to provide insight into the antimicrobial activity of four non-infectious agents, it also is a call upon the scientific community to further evaluate these agents and other similar molecules to aid in the treatment of chronic biofilm infections. Without innovation and development of novel agents to treat infections, patients will continue to be exposed to unabridged morbidity and mortality since conventional antibiotics have limited activity to sessile microbial states. Consequently, the information outlined in this Perspective should spearhead further research into the treatment of chronic biofilm infections with translational and clinical research studies.

## Figures and Tables

**Table 1:** Comparative summary of repurposed non-infectious agents with antibiofilm activity.

Agent	Current Clinical Use	Mechanism of Antimicrobial/Antibiofilm Activity	Effective Concentrations (In Vitro/Ex Vivo)	Target Organisms	Biofilm Stage Affected	Tolerated Clinical Doses	Potential Clinical Applicability	Limitations
Ethyl pyruvate (EP)	Food additive; antioxidant evaluated for ischemia–reperfusion and inflammatory injury [8,9,10,11,12,13,14,15]	Inhibits microbial glycolysis and para-glycolytic pathways, leading to ATP depletion and methylglyoxal accumulation [13,14,17]	MIC as low as 25 mM (~2.9 mg/mL); 50 mM reduced CFUs in preformed biofilms by ~3 logs [17]	*Candida* spp.; Gram-positive and Gram-negative bacteria [17]	Early, Mature [17]	Up to 90 mg/kg in humans [15]	Systemic adjunct for deep-seated or endovascular biofilm infections	Limited human infection data; tissue concentrations not defined
Methylene blue (MB)	FDA approved for methemoglobinemia; intraoperative and diagnostic dye [18,19,20,21]	Photodynamic ROS generation (660 nm) [26]	Enhanced activity with red-light exposure; concentrations depend on organism and light source; 10 nM–10 mM studied [24,25,26,27,28,29]	Gram-positive and Gram-negative bacteria; fungi; viruses [25,26,27,28,29]	Mature [27,28,29]	Up to 4 mg/kg in humans [22]	Local therapy for wounds and intraoperative prosthetic joint infection management	Requires light exposure
Ethylenediaminetetraacetic acid (EDTA)	FDA approved for heavy metal chelation in poisoning; catheter lock therapy; evaluated in cardiovascular disease [30,31,32,33]	Chelates divalent cations destabilizing bacterial membranes and extracellular polymeric substances [34,35,37]	40 mg/mL eradicated biofilm cells after 24 h; 18.6 mg/mL (50 mM) reduced *P. aeruginosa* and *S. aureus* biofilms [34,35,37]	Primarily Gram-negative bacteria; variable Gram-positive activity [34,35,37]	Mature [34,36,38]	Up to 3 g/day in humans [33]	Catheter lock therapy and prolonged local dwell applications	Reduced Gram-positive efficacy; mainly local use
N-acetyl cysteine (NAC)	FDA approved for acetaminophen hepatic toxicity; mucolytic [38,39,40,41,42]	Reduces disulfide bonds, chelates zinc, degrades extracellular DNA, decreases biofilm viscosity, enhances antibiotic penetration [45,46,47,48]	30 mM (~4.9 mg/mL) reduced MRSA growth by ~95%; disrupts mature biofilms [45,46,47,48]	Gram-negative bacteria (*P. aeruginosa*, *K. pneumoniae*, *A. baumannii*); MRSA [45,46,47,48]	Early, Mature [48,49,50]	Up to 3 g/day in humans [43,44,46]	Systemic adjunct to antibiotics for chronic and MDR biofilm infections	Adjunctive; effects quickly reversible after discontinuation

Summary of the mechanisms, effective concentrations, target organisms, biofilm activity, safety data, and potential clinical applications of the four repurposed agents discussed. MIC: minimum inhibitory concentration; FDA: Food and Drug Administration; ROS: reactive oxygen species; MRSA: methicillin-resistant *Staph aureus*.

## Data Availability

Not applicable.
